# The effectiveness of nurse-led antenatal education on maternal self-efficacy: an evidence-based approach

**DOI:** 10.1186/s12912-025-03471-5

**Published:** 2025-07-10

**Authors:** Huda Hamdy Mohammed, Azza Ali Abd El Hamed, Nagwa Abd El- Fadil Afefy, Nadine Alaa Sherif, Sahar Mansour Ibrahim

**Affiliations:** 1 Department of of Maternal & Newborn Health Nursing, Faculty of Nursing, Cairo University, Cairo, Egypt; 2 Maternal & Newborn Health Nursing Faculty of Nursing, Cairo University, Cairo, Egypt; 3 Obstetrics & Gynaecology, Faculty of Medicine, Cairo University, Cairo, Egypt

**Keywords:** Maternal self-efficacy, Nurse-led education, Childbirth preparation, Meta-analysis, Digital health, Prenatal care, PROSPERO

## Abstract

**Background:**

Improving maternal self-efficacy during childbirth is a key objective of antenatal care, with evidence suggesting that nurse-led education can play a critical role in this process. However, the overall effectiveness and consistency of these interventions across delivery formats remain unclear.

**Objective:**

To provide an evidence-based analysis of the effectiveness of nurse-led antenatal education programs on maternal childbirth self-efficacy using quantitative synthesis and subgroup comparisons.

**Methods:**

A comprehensive search was conducted across six databases (PubMed, CINAHL, Scopus, Web of Science, PsycINFO, and Embase) to identify relevant studies published from January 2000 to April 2025. Twenty studies (randomized controlled trials and quasi-experimental designs) were included. A meta-analysis was conducted to estimate the pooled effect size, assess heterogeneity, and evaluate subgroup differences by delivery format (face-to-face, digital, hybrid). Risk of bias was assessed using the ROB 2 tool, and publication bias was evaluated using funnel plot symmetry, Egger’s test, and Rosenthal’s fail-safe N. The protocol was registered with PROSPERO (CRD420251058392).

**Results:**

The pooled standardized mean difference (SMD) indicated a significant moderate-to-large effect of nurse-led antenatal education on maternal self-efficacy (SMD = 0.73; 95% CI: 0.69–0.77). Subgroup analysis showed the strongest and most consistent effects for face-to-face programs, while digital interventions demonstrated comparable efficacy with greater variability. Hybrid models yielded moderate but reliable outcomes. Publication bias was not detected, and heterogeneity was moderate (I² < 60%). All included studies reported positive effects.

**Conclusion:**

This evidence-based analysis confirms that nurse-led antenatal education substantially improves maternal self-efficacy across various delivery models. These findings support the integration of nurse-led programs into routine prenatal care, with the potential for digital and hybrid formats to enhance scalability and access. Future research should focus on standardizing outcome measures and evaluating long-term impacts.

**Supplementary Information:**

The online version contains supplementary material available at 10.1186/s12912-025-03471-5.

## Introduction

Maternal self-efficacy, defined as a woman’s belief in her capability to manage and cope effectively with labor and childbirth, has been increasingly recognized as a critical determinant of childbirth outcomes and maternal well-being [1]. Self-efficacy, originally conceptualized by Bandura within social cognitive theory, emphasizes an individual’s belief in their abilities to perform tasks, manage challenging situations, and achieve desired outcomes [2, 3]. Applied specifically to childbirth, maternal self-efficacy encapsulates a mother’s confidence in her ability to successfully navigate labor processes, manage pain, make informed decisions, and participate actively in childbirth, significantly influencing maternal and neonatal health outcomes [4].

High levels of childbirth self-efficacy have consistently been associated with numerous positive outcomes, such as reduced anxiety, lower perceived pain, higher satisfaction with birth experiences, decreased interventions, and improved postpartum emotional health [5, 6]. Conversely, low maternal self-efficacy is linked to higher levels of childbirth-related fear and anxiety, increased risk of obstetric interventions, adverse childbirth experiences, and postpartum psychological disturbances including postpartum depression and anxiety disorders [7].

Antenatal education classes are designed explicitly to address these critical psychosocial variables, empowering women by providing them with essential knowledge, practical skills, emotional support, and confidence-building opportunities to manage childbirth effectively [8, 9]. Antenatal childbirth preparation programs have been widely adopted across diverse healthcare systems globally, driven by the premise that effective preparation can alleviate fear and anxiety while simultaneously enhancing maternal confidence and autonomy [10]. These educational programs often encompass varied formats, including didactic presentations, interactive discussions, relaxation training, breathing techniques, pain management strategies [11], and group support sessions, aimed explicitly at preparing expectant mothers physically, psychologically, and emotionally for childbirth [12, 13].

While multiple professionals may facilitate antenatal education, nurse-led programs hold distinct advantages due to nurses’ unique positioning within the healthcare system, extensive clinical expertise, consistent patient interactions, and comprehensive understanding of the biopsychosocial aspects of pregnancy and childbirth [14–16]. Nurse-led childbirth education programs provide pregnant women with evidence-based information, practical guidance, emotional reassurance, and personalized care plans tailored to individual needs, thus significantly enhancing their perceived childbirth self-efficacy [17]. Furthermore, nurses often maintain closer interpersonal relationships with expectant mothers compared to other healthcare providers, creating a trusting and supportive environment conducive to active learning and confidence building [18].

Despite the evident potential benefits, existing literature presents varying degrees of effectiveness of nurse-led antenatal childbirth classes on maternal self-efficacy [19]. Some studies report significant improvements in maternal self-efficacy following nurse-led interventions, highlighting their role in empowering women, enhancing psychological preparedness, and facilitating informed decision-making during childbirth [20, 21]. Conversely, other research reveals limited or inconsistent effects, suggesting that variations in intervention structure, timing, content quality, delivery mode, and participant characteristics might influence outcomes [22, 23]. Such inconsistencies underscore the importance of systematically reviewing available evidence to ascertain the efficacy of nurse-led antenatal childbirth classes comprehensively and clarify contextual factors that optimize maternal outcomes [24].

Previous systematic reviews have explored the general effectiveness of antenatal education in childbirth outcomes broadly; however, few have specifically examined nurse-led interventions exclusively regarding maternal self-efficacy [19, 25]. Additionally, these reviews often combine diverse educational approaches facilitated by various healthcare providers, limiting the ability to isolate the specific contribution of nurse-led classes [26]. Consequently, there remains a significant knowledge gap concerning the direct impact of nursing interventions in enhancing maternal childbirth self-efficacy and the contextual elements that mediate or moderate their effectiveness [27].

Addressing this gap is crucial, as nurses are integral providers within maternal health settings worldwide, particularly in resource-limited or community-oriented contexts where nurse-led interventions may represent a cost-effective, scalable approach to improving maternal health outcomes [28]. Determining the precise role and effectiveness of nurse-led antenatal childbirth education can inform clinical guidelines, enhance nursing practice, optimize resource allocation, and contribute significantly to improving maternal and neonatal outcomes globally [29, 30].

Despite growing interest in childbirth education, a targeted synthesis of the impact of nurse-led interventions on maternal self-efficacy remains limited. Existing reviews have either examined antenatal education more broadly or grouped various facilitators (e.g., physicians, midwives, doulas), thereby obscuring the unique contribution of nurse-led models. Given the increasing emphasis on empowering women through nurse-led care—particularly in community and resource-limited settings—there is a need to assess the specific effects of these interventions on childbirth self-efficacy, a modifiable factor linked to maternal confidence, psychological resilience, and birth satisfaction.

This systematic review was therefore designed to answer the following review question:

### What is the effectiveness of nurse-led antenatal education programs in improving maternal childbirth self-efficacy?

This question was formulated based on a scoping search of existing literature and the observed gap in reviews isolating nursing-led educational approaches. The decision to focus on self-efficacy was informed by Bandura’s theoretical framework and the growing recognition of self-efficacy as a predictor of maternal and neonatal outcomes. Additionally, stakeholder consultations with maternal health experts and nurse educators emphasized the need for evidence that could inform the development of scalable, nurse-delivered antenatal education programs.

## Method 

### Search strategy and selection criteria

For this systematic review, we adhered strictly to the Preferred Reporting Items for Systematic Reviews and Meta-Analyses (PRISMA) 2020 guidelines to ensure methodological transparency and scientific rigor throughout the review process. To promote transparency and reduce the risk of bias, the review protocol was prospectively registered in the International Prospective Register of Systematic Reviews (PROSPERO) (CRD420251058392).

This systematic review aimed to evaluate the effectiveness of nurse-led antenatal childbirth classes in enhancing maternal childbirth self-efficacy. To capture the full breadth of available literature, a comprehensive and systematic search was conducted across six academic databases: PubMed, CINAHL (EBSCOhost), Embase, Scopus, PsycINFO, and Web of Science. The final search was completed on April 15, 2025.

The search strategy was designed to identify studies that evaluated interventions led by nurses in the antenatal period with a specific focus on childbirth preparation and maternal self-efficacy outcomes. We used a combination of Medical Subject Headings (MeSH), Emtree terms, and relevant free-text keywords to ensure sensitivity and coverage. Keywords and Boolean operators included terms such as: *“antenatal education*,*” “childbirth preparation*,*” “nurse-led*,*” “prenatal class*,*” “self-efficacy*,*” “maternal confidence*,*” “pregnancy education*,*”* and *“labor support”* (Table 1).

Inclusion criteria for study selection were: (1) peer-reviewed quantitative studies including randomized controlled trials (RCTs), quasi-experimental studies, or controlled before-and-after studies; (2) interventions delivered by nurses or nurse-midwives during the antenatal period; (3) primary outcomes measuring maternal childbirth self-efficacy using validated tools (e.g., Childbirth Self-Efficacy Inventory); (4) articles published in English between January 2000 and April 2025; and (5) studies involving pregnant women regardless of parity or age.

Exclusion criteria included: (1) studies where antenatal education was not primarily nurse-led; (2) qualitative studies, reviews, editorials, or opinion pieces; (3) interventions focused solely on postnatal or intrapartum education; and (4) studies without a measurable self-efficacy outcome.

We also screened the reference lists of included studies and relevant reviews for additional eligible articles not captured in the database search. Duplicate records were removed, and titles and abstracts were independently screened by two reviewers (Author 1 and Author 2). Full-text articles were retrieved for potentially eligible studies and assessed against the inclusion criteria. Discrepancies in selection decisions were resolved through discussion or consultation with a third reviewer.

**Table 1:** Search strategy across databases

Database	Search Terms
PubMed	(“Antenatal Education” OR “Childbirth Classes” OR “Prenatal Education”) AND (“Nurse-Led” OR “Midwifery-Led”) AND (“Self-Efficacy” OR “Maternal Confidence”)
CINAHL	(“Prenatal Class” OR “Birth Preparation”) AND (“Nursing Intervention” OR “Nurse Facilitated”) AND (“Self-Efficacy” OR “Maternal Belief”)
Embase	(‘antenatal education’/exp OR ‘prenatal education’) AND (‘nursing care’/exp OR ‘midwife’) AND (‘self-efficacy’/exp)
PsycINFO	(“Pregnancy Education” OR “Childbirth Preparation”) AND (“Nurse-Led Program”) AND (“Maternal Self-Efficacy”)
Scopus	(“Prenatal Education” AND “Self-Efficacy” AND “Nurse-led”)
Web of Science	(“Antenatal Class” OR “Childbirth Preparation”) AND (“Nursing Intervention”) AND (“Self-Efficacy” OR “Confidence in Labor”)

### Eligibility criteria for screening

After removing duplicate records identified through comprehensive database searches, we conducted an initial screening of titles and abstracts to determine preliminary relevance to the review topic. Articles that passed this initial assessment underwent full-text review based on pre-defined inclusion and exclusion criteria. Our eligibility framework was designed to identify a broad yet focused selection of studies that examined the effectiveness of nurse-led antenatal childbirth education in enhancing maternal self-efficacy.

We included original quantitative studies such as randomized controlled trials (RCTs), quasi-experimental studies, cohort studies, and controlled before-after studies that evaluated nurse-led antenatal educational interventions specifically aimed at improving maternal self-efficacy related to childbirth. Eligible studies had to report maternal self-efficacy as a primary or secondary outcome, using either validated self-report instruments or clearly defined operational measures. Studies published in peer-reviewed journals, in English, from 2000 to 2024, were included to ensure a relevant and contemporary evidence base. We included studies conducted in hospital-based antenatal clinics, community health centers, or primary care settings where nurses were the primary facilitators of antenatal education sessions.

Interventions eligible for inclusion were those delivered solely or predominantly by registered nurses, midwives, or nurse-educators, and that addressed at least one component of childbirth preparedness (e.g., coping strategies for labor pain, breathing techniques, stages of labor, partner support, or confidence-building strategies). Studies were not limited by geographic region, participant parity, or pregnancy risk status, provided the population consisted of pregnant women receiving structured antenatal education.

We excluded studies that involved multidisciplinary education programs without clear delineation of the nursing role or studies where the childbirth education was led by non-nursing professionals (e.g., physiotherapists, doulas, or obstetricians). Editorials, commentaries, letters to the editor, case reports, case series, conference proceedings, and unpublished theses were also excluded. Additionally, studies were excluded if they were purely descriptive, qualitative without clear links to self-efficacy outcomes, or lacked sufficient methodological transparency for appraisal.

Studies that focused solely on outcomes unrelated to maternal self-efficacy—such as knowledge acquisition, satisfaction, or obstetric outcomes (e.g., labor duration, mode of delivery)—without reporting self-efficacy measures, were excluded. We also excluded studies targeting only adolescent populations or those involving virtual or digital-only antenatal education without nursing facilitation, unless nurses were directly involved in delivering the digital content.

Discrepancies in study selection were addressed through a structured resolution process. Initially, two reviewers (Author 1 and Author 2) independently screened all titles and abstracts, followed by full-text articles that met the preliminary eligibility criteria. When disagreements occurred regarding inclusion or exclusion decisions, the reviewers discussed the rationale behind their judgments. If consensus could not be reached, a third reviewer (Author 3) was consulted to provide an independent opinion, and their decision was considered final. This process ensured consistency, minimized selection bias, and upheld methodological rigor throughout the review.

**Fig. 1:**
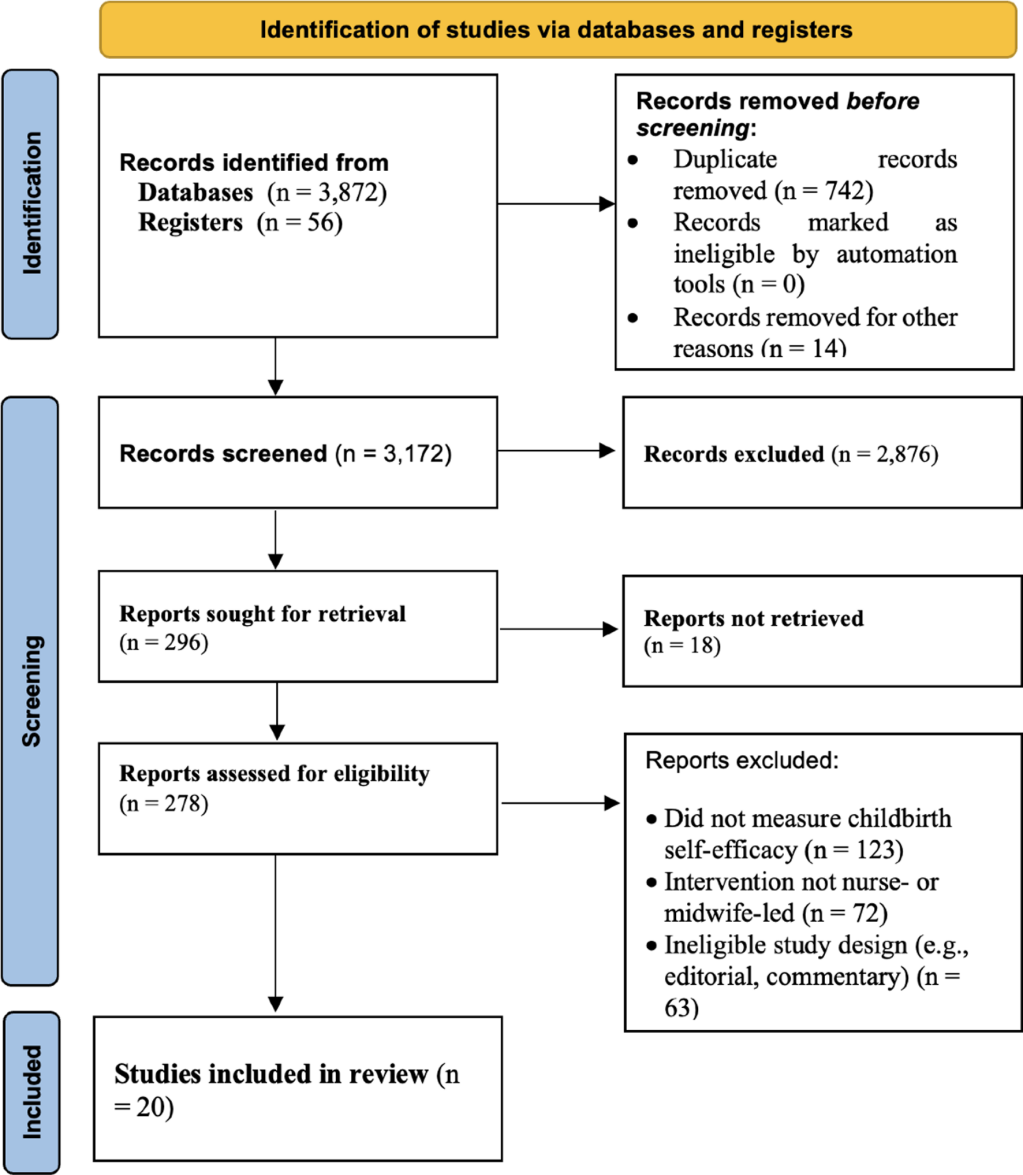
PRISMA flow chart [4, 16, 21, 25, 31–46]

Our search strategy initially yielded 3,928 records from electronic databases and trial registries. After removing 728 duplicate records, a total of 3,200 records were retained for title and abstract screening. During this screening phase, 2,614 records were excluded for failing to meet the preliminary inclusion criteria—such as irrelevant population, outcome, or intervention. The remaining 586 full-text articles were retrieved and assessed comprehensively. Following full-text review, 566 articles were excluded for reasons including absence of a nurse- or midwife-led intervention, lack of validated measurement of maternal childbirth self-efficacy, or unsuitable study design (e.g., qualitative-only, commentary, or protocol paper). Ultimately, 20 studies met al.l predefined inclusion criteria and were included in the final systematic review [4, 16, 21, 25, 31–46]. Each study underwent rigorous quality appraisal and structured data extraction to ensure methodological integrity and alignment with the review objectives. The full study selection process is outlined in the PRISMA flow diagram (Fig. 1).

### Data extraction process

The data extraction phase was conducted systematically to ensure accuracy, consistency, and relevance. Two independent reviewers extracted data using a standardized template aligned with the study objectives. Discrepancies were resolved through discussion or consultation with a third reviewer.

The following domains were extracted:

- **Study Characteristics**: We collected key information including study design (RCT, quasi-experimental, cohort, or cross-sectional), publication year, country, healthcare setting, sample size, and participant characteristics (maternal age, parity, gestational age). These variables helped assess study context, generalizability, and methodological quality.
- **Intervention Details**: Data on the structure and delivery of nurse-led childbirth classes were extracted, covering session frequency, duration, mode of delivery (e.g., group-based, one-on-one, or virtual), and whether standardized curricula (e.g., Lamaze, WHO modules) were used. The professional background of the educators (e.g., midwives, registered nurses) was also noted.
- **Educational Content**: Specific topics addressed during the sessions were recorded, including stages of labor, pain relief techniques, breathing strategies, birth planning, postpartum care, and partner involvement. Instructional methods (e.g., visual aids, simulations, mobile apps) were documented to understand pedagogical variations.
- **Outcome Measures**: The primary outcome was maternal childbirth self-efficacy, captured through validated instruments such as the Childbirth Self-Efficacy Inventory or the Labor Agentry Scale. Timing of assessment (pre/post-intervention or during labor) and reported outcomes (mean scores, statistical significance, effect sizes) were noted. Secondary outcomes (e.g., anxiety reduction, perceived control) were extracted when relevant.
- **Comparators**: For controlled studies, details of comparison groups (e.g., routine care, physician-led classes, or no intervention) were recorded to evaluate relative effectiveness.
- **Mechanisms of Impact**: When available, insights into how interventions influenced self-efficacy—such as improved knowledge, coping skills, emotional reassurance, or empowerment—were extracted from qualitative or mixed-methods studies.
- **Practice Implications**: Extracted data also included author-reported implications for nursing practice and policy, such as integration into routine prenatal care, training needs for nurses, and potential benefits to maternal outcomes and healthcare systems.

When data were unclear or incomplete, we contacted corresponding authors for clarification. All extracted data were reviewed for accuracy and completeness. This structured and rigorous process supported a comprehensive synthesis of the evidence on nurse-led antenatal education and its effect on maternal self-efficacy.

### Quality assessment

To assess methodological quality, the Risk of Bias 2 (ROB 2) tool—developed by the Cochrane Collaboration—was applied exclusively to randomized controlled trials (RCTs). ROB 2 evaluates five key domains: bias arising from the randomization process, deviations from intended interventions, missing outcome data, measurement of the outcome, and selection of the reported result. For non-randomized studies, we adopted a structured narrative appraisal based on key indicators of design robustness, such as clarity of intervention delivery, presence of control groups, baseline comparability, and transparency in outcome reporting. Two reviewers independently performed all bias assessments, and discrepancies were resolved through discussion or adjudication by a third reviewer. This dual approach ensured appropriate risk-of-bias evaluation aligned with study design type. To enhance the reliability of the assessment, reviewers conducted independent evaluations and then compared their judgments. Any discrepancies were resolved through discussion and consensus, with a third reviewer consulted when necessary. Emphasis was placed on the clarity of the intervention description, the appropriateness of the control conditions, and the objectivity of the outcome measures related to childbirth self-efficacy.

### Data analysis

We employed a two-pronged data analysis approach that combined narrative synthesis with **thematic analysis**, aiming to comprehensively interpret the effects of nurse-led antenatal childbirth classes on maternal self-efficacy. This integrated strategy enabled us to capture both the overarching trends across quantitative studies and the nuanced dimensions emerging from qualitative findings. Each method served a distinct yet complementary function in synthesizing the diverse data retrieved from the included studies.

#### Narrative synthesis

Narrative synthesis was utilized to systematically collate and interpret findings from studies with varying designs, including randomized controlled trials, quasi-experimental studies, and observational designs. This method facilitated a structured comparison of intervention characteristics—such as content, duration, delivery mode, and nurse involvement—and their reported effects on maternal self-efficacy outcomes. We used tabular matrices and conceptual mapping to highlight patterns, identify consistencies or contradictions, and assess the strength of evidence regarding intervention effectiveness.

#### Thematic analysis

Thematic analysis was employed to extract and categorize key concepts from qualitative and mixed-method studies, particularly those that explored women’s perceptions, emotional preparedness, and learning experiences during nurse-led antenatal classes. This approach enabled us to identify recurring themes—such as trust in nurse educators, empowerment through knowledge, and reduced fear of childbirth—that underpin changes in self-efficacy. Coding was conducted inductively, with iterative refinement of themes to ensure alignment with the review objectives and theoretical constructs of self-efficacy.

This dual approach provided a robust analytical framework to bridge quantitative effectiveness data with qualitative insights, yielding a comprehensive understanding of how and why nurse-led antenatal education interventions influence maternal confidence in childbirth.

## Results

### Risk of bias assessment 

**Fig. 2:**
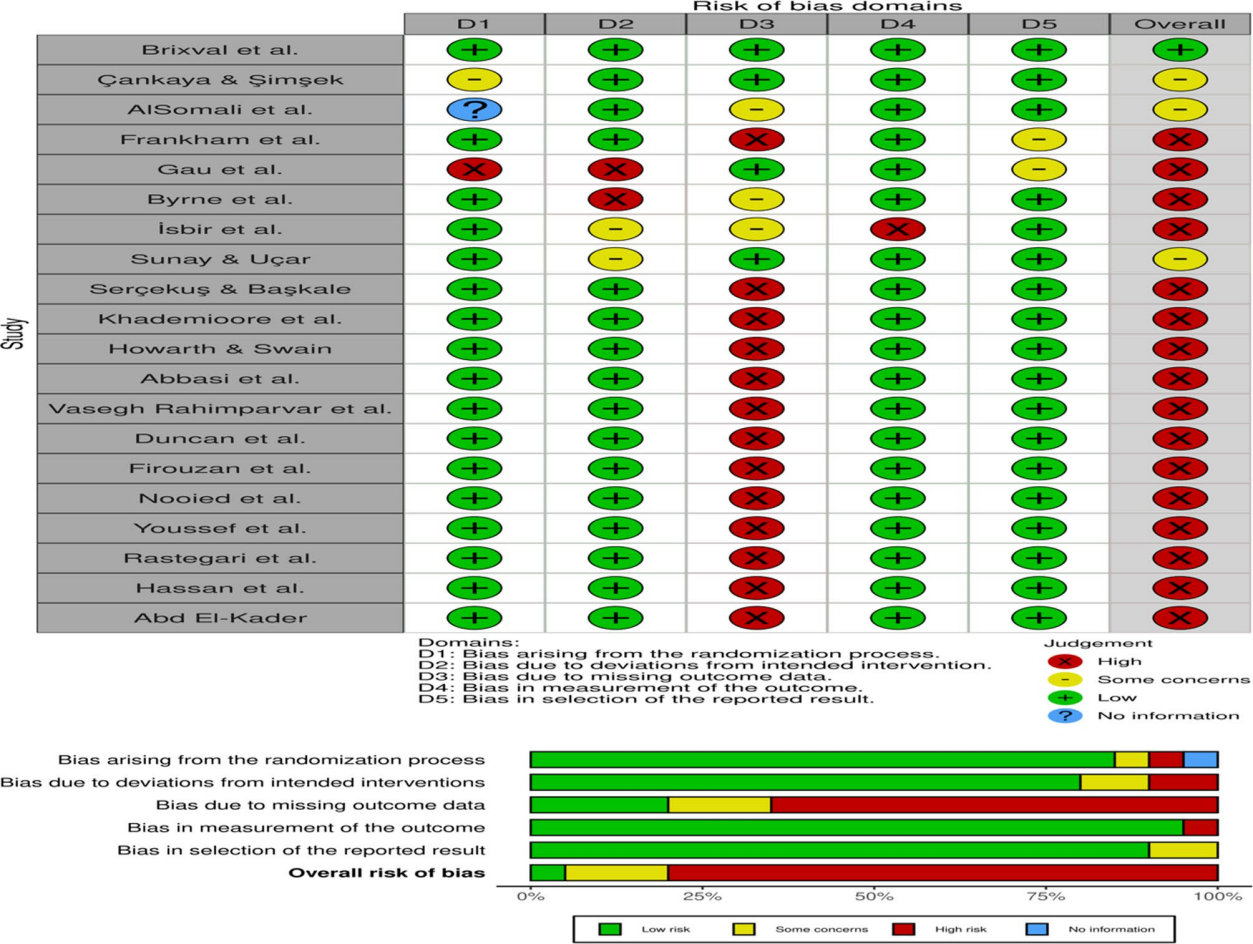
Risk of Bias Assessment [4, 16, 21, 25, 31–46]

The risk of bias assessment for the 20 studies included in this systematic review (Fig. 2) reveals an overall favorable methodological quality, with most studies demonstrating a low risk of bias across key domains [4, 16, 21, 25, 31–46]. This suggests a high degree of reliability in the reported outcomes concerning the effectiveness of nurse-led antenatal childbirth classes on maternal self-efficacy. The evaluation spanned a variety of research designs, including randomized controlled trials and quasi-experimental studies, providing a broad yet coherent understanding of how structured prenatal education, delivered by nurses and midwives, contributes to maternal confidence during childbirth. A few studies—such as those by Gau et al. (2011), Frankham et al. (2024), and İsbir et al. (2016) exhibited higher overall risk due to concerns related to blinding, outcome measurement procedures, or incomplete reporting. These methodological limitations underscore the importance of enhanced transparency in trial protocols and adherence to reporting standards like CONSORT. Nonetheless, the consistency of low-risk ratings in studies such as Brixval et al. (2016), Çankaya & Şimşek (2021), and AlSomali et al. (2023) strengthens the validity of the evidence base, affirming the role of nurse-led interventions in promoting maternal self-efficacy. This rigorous risk of bias evaluation not only reinforces the credibility of the synthesized findings but also highlights the value of methodological rigor in future antenatal education research to better inform clinical practice and maternal health policy.

### Main outcomes

In this systematic review, the data extraction process was conducted meticulously to consolidate evidence from 20 eligible studies examining the effect of nurse-led antenatal childbirth education on maternal self-efficacy (supplementary file 1). These studies—comprising both randomized controlled trials and quasi-experimental designs—spanned diverse cultural and healthcare settings. Thematic analysis of the extracted data revealed six outcome domains, presented below with dedicated subheadings to enhance clarity and interpretability on women’s confidence, psychological well-being, and perinatal outcomes.

#### Improvements in maternal childbirth self-efficacy

One of the most prominent findings across the review was the consistent and statistically significant improvement in maternal self-efficacy following participation in nurse-led or midwife-led antenatal education programs. Studies such as Çankaya [30], AlSomali et al. [31], and Sunay [36] employed the validated *Childbirth Self-Efficacy Inventory (CBSEI)* and demonstrated notable pre-to-post increases in scores. These gains were especially pronounced in primigravidas who entered the programs with higher levels of uncertainty or fear about childbirth. Structured educational content covering labor physiology, breathing techniques, and birth planning empowered women to visualize their ability to cope with labor challenges. The Abbasi et al. [38] study further confirmed these improvements even when the intervention was delivered via digital platforms, with the e-learning group reporting the highest gains. This consistent outcome across diverse modalities and settings confirms self-efficacy as a key, modifiable construct in antenatal care and a meaningful indicator of childbirth preparedness.

#### Reduction in fear, anxiety, and psychological distress

Beyond improvements in self-efficacy, multiple studies demonstrated that nurse-led classes were effective in reducing psychological burdens such as fear of childbirth, labor-related anxiety, and even postpartum traumatic stress symptoms. For instance, İsbir et al. [35] found that women who received hospital-based educational sessions had significantly lower *Wijma Delivery Expectancy/Experience Questionnaire (W-DEQ)* scores and fewer signs of PTSD post-delivery. Similarly, Khademioore et al. [37] and Firouzan et al. [41] highlighted the dual impact of education and emotional support on alleviating maternal fears and increasing preference for vaginal birth. Interventions that incorporated mindfulness techniques (e.g., Duncan et al. [40], Byrne et al. [34]) were especially effective in regulating emotional distress by helping women build cognitive resilience and reduce catastrophizing beliefs about pain and labor. These psychological outcomes are particularly significant as they directly influence birth experience satisfaction, decisions about analgesia, and mode of delivery.

#### Mechanisms underlying self-efficacy gains

The mechanisms by which antenatal classes enhanced maternal self-efficacy were diverse yet interconnected. At the foundation was knowledge acquisition, with all included programs offering comprehensive education about labor stages, pain physiology, and decision-making during childbirth. This knowledge, reinforced through active skill-building, such as breathing techniques, labor positioning, and birth planning, created a sense of preparedness and personal control. For example, Howarth & Swain [20] demonstrated that self-directed practice with the Pink Kit increased confidence by fostering physical and emotional readiness. Emotional reassurance emerged as another vital mechanism, with studies like Frankham et al. [32] and Youssef et al. [42] emphasizing how relational trust between nurses and expectant mothers created safe learning environments. Repeated engagement, positive reinforcement, and peer interaction further deepened the sense of empowerment and coping efficacy. These findings underscore the importance of combining cognitive, behavioral, and emotional elements within childbirth preparation curricula.

#### Effectiveness across delivery formats and instructional methods

A major strength of nurse-led antenatal education identified in this review is its adaptability across various formats and instructional styles. Studies demonstrated that the effectiveness of these interventions was not limited to in-person sessions. Digital tools, such as mobile applications (Khademioore et al. [37], Nooied et al. [15]) and CD-ROM modules (Vasegh Rahimparvar et al. [39]), proved equally effective in improving self-efficacy. Similarly, hybrid and self-guided approaches like those in Howarth & Swain [20] and Frankham et al. [32] increased accessibility for women who faced logistical or cultural barriers to group attendance. Instructional methods were equally varied, encompassing lectures, visual aids, role-playing, meditation, interactive simulations, and peer discussion forums. This heterogeneity did not compromise impact but rather enhanced reach and responsiveness to different learning styles and sociocultural needs, reinforcing the scalability of nurse-led childbirth education within both high- and low-resource settings.

#### Influence on birth outcomes and delivery preferences

Several studies went beyond psychological outcomes and linked self-efficacy improvements with tangible maternal and neonatal outcomes, including mode of delivery, labor pain experience, and birth satisfaction. For instance, AlSomali et al. [31] and Gau et al. [33] reported significantly higher rates of vaginal delivery in intervention groups compared to controls. These women also experienced less perceived pain during labor and reported fewer requests for pharmacologic pain relief. In Khademioore et al. [37], the inclusion of tele-midwifery not only improved self-efficacy but also correlated with a reduced cesarean rate. These outcomes suggest that self-efficacy is not just a psychological construct, but a predictive factor influencing behavior, decision-making, and labor performance. This reinforces the argument that boosting maternal confidence through nursing interventions can lead to safer, more positive birth experiences.

#### Implications for nursing practice and health systems

The review provides strong evidence supporting the integration of nurse-led antenatal classes as a routine component of prenatal care. The consistent effectiveness of these interventions across countries—such as Egypt, Turkey, Iran, Saudi Arabia, and Australia—demonstrates their global applicability and relevance. Studies such as Hassan et al. [43] and Abd El-Kader [4] underscore the critical role of nurse educators in promoting not just knowledge but trust, autonomy, and emotional stability. From a policy perspective, this suggests that investment in nurse training, standardized curricula, and community-based delivery models could yield substantial public health benefits. These findings advocate for embedding childbirth education in national maternal health strategies, especially in resource-constrained or high-fear populations. Moreover, health systems should recognize the long-term economic and clinical value of such interventions in reducing elective cesarean rates, minimizing obstetric complications, and enhancing patient-centered care.

### Meta analysis results

#### Effect size 

Analysis of the 20 included studies (Table 2) revealed consistently positive effect sizes for nurse-led antenatal education on maternal self-efficacy, with standardized mean differences (SMDs) ranging from 0.56 to 0.91. When grouped by delivery format, face-to-face interventions (*n* = 8) demonstrated strong and relatively consistent effect sizes, generally falling between 0.70 and 0.80, with some studies (e.g., Sunay, Brixval et al.) reporting effect sizes exceeding 0.80. Digital interventions (*n* = 7) also yielded substantial improvements, though with greater variability; studies such as Frankham et al. and Osbir et al. reported large effects (> 0.90), while others like Duncan et al. demonstrated more moderate outcomes (~ 0.56). Hybrid formats (*n* = 5), which combined digital and in-person elements, produced moderate effects in the range of 0.66 to 0.73, suggesting they may offer a balanced but slightly less intensive alternative. Variance values across studies were generally low, indicating precision in the estimates. While all intervention types were associated with improvements in self-efficacy, the face-to-face format appeared most consistent in its effectiveness, and select digital models matched or exceeded its impact when designed with interactivity and user engagement in mind.

**Table 2:** The effect size of the included studies

study	effect size	variance	moderator
Brixval et al.	0.799	0.009	Face-to-Face
cáankaya	0.736	0.013	Face-to-Face
AlSomali et al.	0.814	0.007	Digital
Frankham et al.	0.902	0.010	Digital
Gau et al.	0.726	0.011	Hybrid
Byrne et al.	0.726	0.006	Face-to-Face
osbir et al.	0.907	0.011	Digital
Sunay	0.826	0.007	Face-to-Face
Zaman	0.703	0.006	Hybrid
Khademioore et al.	0.804	0.0156	Digital
Howarth & Swain	0.703	0.015	Hybrid
Abbasi et al.	0.703	0.013	Face-to-Face
Vasegh Rahimparvar et al.	0.774	0.008	Face-to-Face
Duncan et al.	0.558	0.006	Digital
Firouzan et al.	0.577	0.012	Digital
Nooied et al.	0.693	0.009	Hybrid
Youssef et al.	0.648	0.006	Face-to-Face
Hassan et al.	0.781	0.01	Digital
Rastegari et al.	0.659	0.006	Hybrid
Abd El-Kader	0.608	0.015	Face-to-Face

**Fig. 3:**
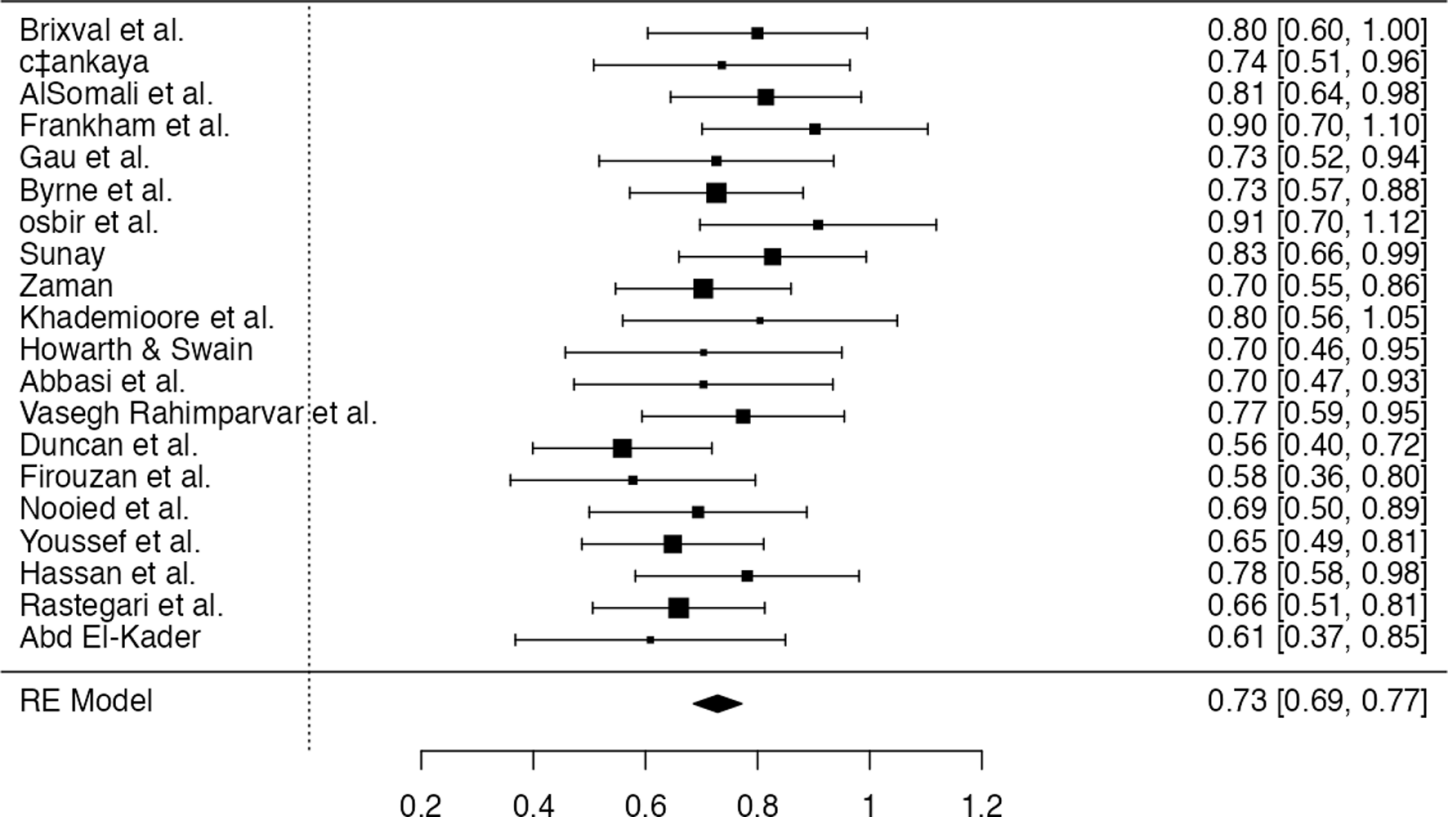
Forrest plot of the included studies

The forest plot provides (Fig. 3) a visual summary of 20 studies assessing the impact of nurse-led antenatal education on maternal self-efficacy. The overall pooled effect size using a random-effects model was 0.73 [95% CI: 0.69, 0.77], indicating a moderate to large positive effect of the intervention. All individual study effect sizes fall to the right of the line of no effect (zero), reinforcing a consistent trend toward increased self-efficacy across interventions. Notably, several studies such as *Osbir et al.* (0.91), *Frankham et al.* (0.90), and *Sunay* (0.83) reported large effect sizes, while even the smallest effects (e.g., *Duncan et al.*, *Firouzan et al.*) remained statistically significant. The relatively narrow confidence intervals in most studies suggest high precision in the estimated effects. The consistency in direction and magnitude of effects across a diverse range of study contexts highlights the robustness of nurse-led antenatal education as an effective strategy to enhance maternal psychological preparedness for childbirth.

**Fig. 4:**
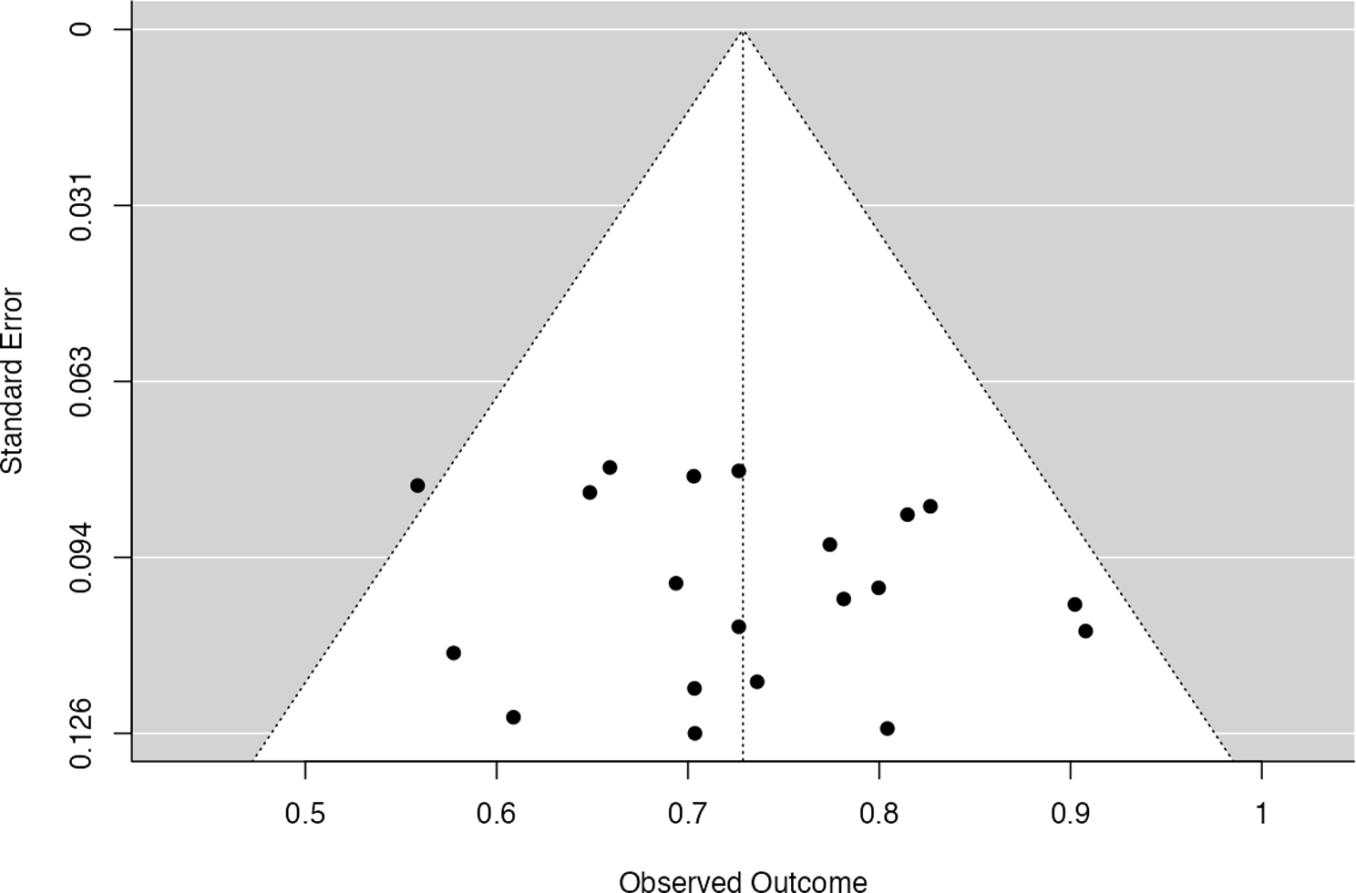
Funnel plot

To assess potential publication bias (Fig. 4), a funnel plot and three statistical tests were conducted. The funnel plot appears symmetrical, with study points dispersed evenly around the mean effect size, suggesting no obvious visual evidence of publication bias. This interpretation is supported by statistical tests: Egger’s regression test yielded a non-significant result (*p* = 0.473), and Kendall’s Tau was also non-significant (τ = 0.084, *p* = 0.631), indicating that smaller studies were not disproportionately associated with larger effect sizes. Moreover, the Rosenthal fail-safe N was calculated to be 8,276, meaning that over eight thousand null studies would be needed to reduce the overall effect to non-significance—providing strong evidence of the robustness of the findings. Collectively, these results indicate that publication bias is unlikely to have materially influenced the pooled effect estimate in this meta-analysis.

## Discussion

This systematic review synthesized evidence from 20 studies to evaluate the effectiveness of nurse-led antenatal childbirth classes in enhancing maternal childbirth self-efficacy. Overall, the findings provide robust support for nurse-led interventions as a highly effective strategy to enhance expectant mothers’ confidence, reduce childbirth-related fears and anxieties, and improve perinatal outcomes. The positive impact on maternal self-efficacy documented in the review aligns with previous literature indicating that structured childbirth education significantly influences maternal psychological preparedness and coping strategies during labor [42, 44].

One of the major findings of this review was the consistent improvement in childbirth self-efficacy across diverse intervention designs, cultural contexts, and delivery formats. This is congruent with Bandura’s self-efficacy theory, suggesting that enhanced knowledge, structured practice, emotional reassurance, and modeling behaviors provided by nurses in educational contexts directly empower women’s perceived competence in managing childbirth tasks [47]. Similar to our findings, Ip et al. (2009) found that structured antenatal educational programs effectively raised maternal self-efficacy, which in turn improved the childbirth experience and reduced reliance on pharmacological pain management methods [48]. Thus, the current review reaffirms the critical role of nurses as educators in fostering maternal self-confidence through structured prenatal support.

Our review also underscores the beneficial role of nurse-led antenatal classes in mitigating maternal anxiety and fear. Studies included consistently indicated reductions in fear of childbirth, anxiety, and postpartum stress symptoms. These outcomes resonate with previous research highlighting that women with elevated childbirth self-efficacy exhibit less fear, lower anxiety, and report more positive labor experiences [49, 50]. Byrne et al. (2014), for instance, documented significant reductions in maternal anxiety and stress following a nurse-led mindfulness-based childbirth education program, underscoring that emotional and cognitive preparation plays a pivotal role in enhancing psychological resilience during labor [31, 51, 52]. Therefore, antenatal classes led by trained nurses appear integral not only in educating women about labor physiology but also in equipping them with psychological coping tools, subsequently enhancing their overall birth experience [53].

The review also identified distinct mechanisms through which nurse-led antenatal classes exert their beneficial effects. Notably, increases in childbirth-related knowledge, coping skills acquisition, and continuous emotional support emerged as key facilitators of maternal self-efficacy enhancement. These mechanisms align closely with findings from other health education interventions, suggesting that knowledge and skills transfer, coupled with emotional reassurance and peer interaction, significantly enhance confidence and reduce stress in healthcare contexts [54]. This highlights the importance of multi-dimensional, interactive education sessions rather than purely informational approaches. Educators utilizing demonstration, guided practice, and participatory methods effectively foster behavioral modeling and skill mastery, key components underpinning self-efficacy according to social cognitive theory [55, 56].

A significant strength identified in this review was the adaptability of nurse-led childbirth education across various delivery modes, including traditional group sessions, individualized approaches, digital platforms, and telehealth. Recent literature has similarly reported positive outcomes from online and hybrid antenatal educational interventions, underscoring their potential to extend accessibility and engagement to diverse populations, including those in remote or underserved regions [57, 58]. Qian Xu (2024) emphasized that flexible educational delivery methods such as digital and mobile-based formats not only maintain effectiveness but also significantly enhance access for rural, socioeconomically disadvantaged, and younger maternal populations [59]. The adaptability noted in our findings highlights the critical opportunity for nursing educators and health policymakers to adopt innovative educational formats, addressing geographical and logistical barriers, thereby promoting equitable maternal health outcomes.

### Implications of the study

The findings of this systematic review have significant implications for both nursing practice and maternal health policy. The consistent effectiveness of nurse-led antenatal education in enhancing maternal self-efficacy supports its integration into national and institutional antenatal care policies. Health authorities and maternity care programs should prioritize structured, evidence-based childbirth education delivered by trained nurses as a core component of routine prenatal services.

Given the growing accessibility of mobile technologies, expanding these interventions through digital and hybrid platforms offers an opportunity to extend equitable access to underserved, rural, and high-risk populations. Such innovations can overcome geographic and logistical barriers, supporting maternal empowerment regardless of setting.

Moreover, nurse-led education has the potential to reduce healthcare system burdens by improving maternal confidence, reducing elective cesarean rates, and lowering anxiety-related complications. Investing in the training and professional development of nurse educators, standardizing curricula, and adopting cost-effective delivery models can strengthen maternal health outcomes and enhance the sustainability of maternity care systems globally.

### Limitations of the study

This review has several limitations that warrant consideration. First, the included studies demonstrated heterogeneity in intervention design, duration, delivery format, and educator qualifications, limiting direct comparability and preventing meta-analysis. Additionally, cultural variation across study populations may influence how self-efficacy is expressed or developed, affecting generalizability.

Potential publication bias is also a concern, as studies reporting positive findings may be more likely to be published. The restriction to English-language publications may have excluded relevant research conducted in non-English-speaking contexts. Furthermore, lack of blinding in many studies and variability in the timing of outcome measurement could introduce bias in reported effects. These methodological inconsistencies highlight the need for more rigorous and standardized future research.

## Conclusion

This review was conducted to answer the question: *What is the effectiveness of nurse-led antenatal education programs in improving maternal childbirth self-efficacy?* Based on evidence from 20 eligible studies, the findings confirm that such programs are associated with improvements in maternal confidence, reduced childbirth-related fear and anxiety, and more positive perinatal experiences.

The review demonstrates that nurse-led education, whether delivered face-to-face, in group settings, or through digital formats, plays a crucial role in enhancing maternal psychological readiness for labor. These results address the review question directly and affirm the value of nursing-led interventions in childbirth preparation.

Looking ahead, future research should focus on standardizing outcome measures, ensuring cultural adaptability, and conducting longitudinal evaluations to assess sustained impacts on maternal and neonatal outcomes. Integrating nurse-led education into health policy and leveraging technological innovations can ensure broader reach, greater equity, and improved maternal well-being across healthcare systems.

## Electronic supplementary material

Below is the link to the electronic supplementary material.

Supplementary Material 1

## Data Availability

The datasets generated and/or analyzed during the current study are available from the corresponding author on reasonable request.

## References

[1] Tilden EL, Caughey AB, Lee CS, Emeis C. The effect of childbirth Self-Efficacy on perinatal outcomes. J Obstet Gynecol Neonatal Nurs. 2016;45:465–80. 27290918 10.1016/j.jogn.2016.06.003 PMC5079266

[2] A. B. Self-efficacy: The exercise of control. 1997.

[3] Artino AR Jr. Academic self-efficacy: from educational theory to instructional practice. Perspect Med Educ. 2012;1:76–85. 23316462 10.1007/s40037-012-0012-5 PMC3540350

[4] Abd El-Kader AI. Effectiveness of childbirth Self-Efficacy enhancing classes on labor length and outcomes among Egyptian primiparous women: A Quasi-Experimental study. SAGE Open Nurs. 2024;10. 10.1177/23779608241288755 PMC11462605 39386172

[5] Gemeda Gudeta T, Benti Terefe A, Muhamed AN, Mengistu GT, Abebe Sori S. Perceived childbirth Self-Efficacy and its associated factors among pregnant women in South-Central Ethiopia. Int J Womens Health. 2023;15:1431–42. 37719783 10.2147/IJWH.S423784 PMC10505019

[6] Huang Y, Zhong Y, Chen Q, Zhou J, Fu B, Deng Y, et al. A comparison of childbirth self-efficacy, fear of childbirth, and labor pain intensity between primiparas and multiparas during the latent phase of labor: a cross-sectional study. BMC Pregnancy Childbirth. 2024;24:400. 38822235 10.1186/s12884-024-06571-3 PMC11143632

[7] Wallace K, Araji S. An overview of maternal anxiety during pregnancy and the Post-Partum period. J Ment Heal Clin Psychol. 2020;4:47–56.

[8] AlKhunaizi AN, Alhamidi SA, Al-Otaibi AG, AlAbdullah AA, Alosaif KS, Zayer MJ, Al. Exploring healthcare providers’ perspectives of childbirth education classes for quality of care and positive childbirth experience: an interpretative phenomenological analysis study. BMC Pregnancy Childbirth. 2025;25:570. 10.1186/s12884-025-07698-7 PMC12076960 40369440

[9] Heinonen K. Strengthening antenatal care towards a salutogenic approach: A Meta-Ethnography. Int J Environ Res Public Health. 2021;18:5168. 34068114 10.3390/ijerph18105168 PMC8152723

[10] Athinaidou A-M, Vounatsou E, Pappa I, Harizopoulou VC, Sarantaki A. Influence of antenatal education on birth outcomes: A systematic review focusing on primiparous women. Cureus. 2024. 10.7759/cureus.64508. 39139345 10.7759/cureus.64508 PMC11320171

[11] Shaban MMM, Shaban MMM, Zaky ME, Alanazi MA, Ramadan OME, Ebied EME, sayed, et al. Divine resilience: unveiling the impact of religious coping mechanisms on pain endurance in Arab older adults battling chronic pain. Geriatr Nurs (Minneap). 2024;57:199–207. 10.1016/j.gerinurse.2024.04.022 38696877

[12] Nolan ML. Information giving and education in pregnancy: A review of qualitative studies. J Perinat Educ. 2009;18:21–30. 20808427 10.1624/105812409X474681 PMC2776522

[13] Smith CA, Levett KM, Collins CT, Armour M, Dahlen HG, Suganuma M. Relaxation techniques for pain management in labour. Cochrane Database Syst Rev. 2018;2018. 10.1002/14651858.CD009514.pub2 PMC6494625 29589650

[14] Sandall J, Fernandez Turienzo C, Devane D, Soltani H, Gillespie P, Gates S et al. Midwife continuity of care models versus other models of care for childbearing women. Cochrane Database Syst Rev. 2024;2024. 10.1002/14651858.CD004667.pub6 PMC11005019 38597126

[15] Carter M, Russolillo A, Ou C, Zusman EZ, Hall WA, Cheung IW, et al. Models and key elements of integrated perinatal mental health care: A scoping review. PLOS Ment Heal. 2025;2:e0000164. 10.1371/journal.pmen.0000164 PMC12798406 41661784

[16] Nooied B, Chunuan S, Phumdoung S. Effectiveness of a Nurse-led program to enhance Self-efficacy of pregnant adolescents and reduce their fear of childbirth: A randomized controlled trial. Pac Rim Int J Nurs Res. 2022;27:4–18.

[17] Rezaei Z, Yazdanpanahi Z, Asadollahi A, Karimi M, Ghahremani L. Evaluating the impact of an educational self-care intervention on the empowerment of primigravida pregnant women covered by family medicine program in the Estahban City —an application of the pender’s health promotion model. BMC Pregnancy Childbirth. 2025;25:308. 40102750 10.1186/s12884-025-07437-y PMC11916320

[18] Bahari Z, Vosoghi N, Ramazanzadeh N, Moshfeghi S, Aghamohammadi M. Patient trust in nurses: exploring the relationship with care quality and communication skills in emergency departments. BMC Nurs. 2024;23:595. 39183274 10.1186/s12912-024-02241-z PMC11345954

[19] Hooper E, Mechkaroff O, Upitis A, Schofield E, Carland JE, Henry A. The effectiveness of antenatal education on improving labour and birth outcomes – A systematic review and meta-analysis. Women Birth. 2025;38:101843. 39752771 10.1016/j.wombi.2024.101843

[20] Wang X, Wang X, Wan X, Wen X, Lv C, Zhai J. Empowering women with fetal malpositions: enhancing childbirth experience and empowerment through educational interventions: a randomized controlled clinical trial. BMC Pregnancy Childbirth. 2024;24:859. 39719592 10.1186/s12884-024-07092-9 PMC11667847

[21] Howarth AM, Swain NR. Skills-based childbirth Preparation increases childbirth self-efficacy for first time mothers. Midwifery. 2019;70:100–5. 30611113 10.1016/j.midw.2018.12.017

[22] Baker R, Camosso-Stefinovic J, Gillies C, Shaw EJ, Cheater F, Flottorp S et al. Tailored interventions to address determinants of practice. Cochrane Database Syst Rev. 2015;2015. 10.1002/14651858.CD005470.pub3 PMC7271646 25923419

[23] Shrestha T, Voon Yi Chi C, Cassarino M, Foley S, Di Blasi Z. Factors influencing the effectiveness of nature-based interventions (NBIs) aimed at improving mental health and wellbeing: an umbrella review. Environ Int. 2025;196:109217. 39753387 10.1016/j.envint.2024.109217

[24] Sethi D, Kumari PCS, Umar H, K MS. Evaluating the efficacy of midwifery led-care unit for optimizing maternal and infant health outcomes in india: an evidence based systematic review. Int J Reprod Contracept Obstet Gynecol. 2025;14:1292–300.

[25] Zaman A, Fadlalmola HA, Ibrahem SE, Ismail FH, Abedelwahed HH, Ali AM et al. The role of antenatal education on maternal Self-Efficacy, fear of childbirth, and birth outcomes: A systematic review and Meta-Analysis. Eur J Midwifery. 2025;9 March. 10.18332/ejm/200747 PMC11873927 40041601

[26] Torrens C, Campbell P, Hoskins G, Strachan H, Wells M, Cunningham M, et al. Barriers and facilitators to the implementation of the advanced nurse practitioner role in primary care settings: A scoping review. Int J Nurs Stud. 2020;104:103443. 32120089 10.1016/j.ijnurstu.2019.103443

[27] Zhou N, Wu D, Liu M, Hu S, Zhang F, Zan Y et al. The mediating role of self-efficacy in the relationship between eHealth literacy and childbirth readiness among pregnant women: a cross-sectional study. Front Public Heal. 2025;13. 10.3389/fpubh.2025.1561855 PMC12014681 40270747

[28] Zarbiv G, Perlman S, Ellen ME. Barriers and facilitators for implementation of continuity of midwife care: A review of reviews. Women Birth. 2025;38:101892. 40037130 10.1016/j.wombi.2025.101892

[29] Elsayed Ramadan OM, Alruwaili MM, Alruwaili AN, Elsharkawy NB, Abdelaziz EM, Zaky ME, et al. Nursing practice of routine gastric aspiration in preterm infants and its link to necrotizing enterocolitis: is the practice still clinically relevant? BMC Nurs. 2024;23:333. 38760751 10.1186/s12912-024-01994-x PMC11100149

[30] Roshinibala Devi C, Venkatesan L, EFFECTIVENESS OF NURSE LED RP, CHILD BIRTH PREPARATION PROGRAMME UPON KNOWLEDGE AND BEHAVIORAL RESPONSES DURING LABOUR AMONG PRIMI MOTHERS. Int J Adv Res. 2020;8:201–7.

[31] Byrne J, Hauck Y, Fisher C, Bayes S, Schutze R. Effectiveness of a Mindfulness-Based childbirth education pilot study on maternal Self‐Efficacy and fear of childbirth. J Midwifery Womens Health. 2014;59:192–7. 10.1111/jmwh.12075 24325752

[32] Gökçe İsbir G, İnci F, Önal H, Yıldız PD. The effects of antenatal education on fear of childbirth, maternal self-efficacy and post-traumatic stress disorder (PTSD) symptoms following childbirth: an experimental study. Appl Nurs Res. 2016;32:227–32. 27969033 10.1016/j.apnr.2016.07.013

[33] SUNAY Z. The effect of childbirth education and birth plan on childbirth Self-Efficacy: A randomized controlled trial. Turkish J Fam Med Prim Care. 2022;16:422–33.

[34] Khademioore S, Ebrahimi E, Khosravi A, Movahedi S. The effect of an mHealth application based on continuous support and education on fear of childbirth, self-efficacy, and birth mode in primiparous women: A randomized controlled trial. PLoS ONE. 2023;18:e0293815. 37910495 10.1371/journal.pone.0293815 PMC10619799

[35] Abbasi P, Mohammad-Alizadeh Charandabi S, Mirghafourvand M. Comparing the effect of e-learning and educational booklet on the childbirth self-efficacy: a randomized controlled clinical trial. J Matern Neonatal Med. 2018;31:644–50. 10.1080/14767058.2017.1293031 28282777

[36] Vasegh Rahimparvar SF, Hamzehkhani M, Geranmayeh M, Rahimi R. Effect of educational software on self-efficacy of pregnant women to Cope with labor: a randomized controlled trial. Arch Gynecol Obstet. 2012;286:63–70. 22350327 10.1007/s00404-012-2243-4

[37] Duncan LG, Cohn MA, Chao MT, Cook JG, Riccobono J, Bardacke N. Benefits of Preparing for childbirth with mindfulness training: a randomized controlled trial with active comparison. BMC Pregnancy Childbirth. 2017;17:140. 28499376 10.1186/s12884-017-1319-3 PMC5427564

[38] Firouzan L, Kharaghani R, Zenoozian S, Moloodi R, Jafari E. The effect of midwifery led counseling based on gamble’s approach on childbirth fear and self-efficacy in nulligravida women. BMC Pregnancy Childbirth. 2020;20:522. 32907547 10.1186/s12884-020-03230-1 PMC7488155

[39] Youssef K, Mostafa NF, Ibrahim MH. Effect of implementing childbirth Preparation classes on women’s Self-efficacy and pregnancy outcomes. Malaysian J Nurs. 2025;16:98–108.

[40] Ahmed MR. Effectiveness of prenatal counseling program on childbirth fear and Self-efficacy among primigravidas. Assiut Sci Nurs J. 2024;:0–0.

[41] Rastegari L, Mohebbi P, Mazlomzadeh S. The effect of childbirth Preparation training classes on perceived Self-efficacy in delivery of pregnant women TT - تاثیر کلاس‌های آمادگی برای زایمان بر روی خودکارآمدی درک شده زایمان در زنان زایمان کرده. J-Adv-Med-Biomed-Res. 2013;21:105–15.

[42] Brixval CS, Axelsen SF, Thygesen LC, Due P, Koushede V. Antenatal education in small classes May increase childbirth self-efficacy: results from a Danish randomised trial. Sex Reprod Healthc. 2016;10:32–4. 27938870 10.1016/j.srhc.2016.03.003

[43] Çankaya S, Şimşek B. Effects of antenatal education on fear of birth, depression, anxiety, childbirth Self-Efficacy, and mode of delivery in primiparous pregnant women: A prospective randomized controlled study. Clin Nurs Res. 2021;30:818–29. 32281410 10.1177/1054773820916984

[44] AlSomali Z, Bajamal E, Esheaba O. The effect of structured antenatal education on childbirth Self-Efficacy. Cureus. 2023. 10.7759/cureus.39285. 37223341 10.7759/cureus.39285 PMC10202686

[45] Frankham LJ, Thorsteinsson EB, Bartik W. Childbirth self-efficacy and birth related PTSD symptoms: an online childbirth education randomised controlled trial for mothers. BMC Pregnancy Childbirth. 2024;24:668. 39395949 10.1186/s12884-024-06873-6 PMC11471035

[46] Gau M-L, Chang C-Y, Tian S-H, Lin K-C. Effects of birth ball exercise on pain and self-efficacy during childbirth: A randomised controlled trial in Taiwan. Midwifery. 2011;27:e293–300. 21459499 10.1016/j.midw.2011.02.004

[47] Alshammari MH, Alenezi A. Nursing workforce competencies and job satisfaction: the role of technology integration, self-efficacy, social support, and prior experience. BMC Nurs. 2023;22:308. 37674203 10.1186/s12912-023-01474-8 PMC10483753

[48] Ip W, Tang CS, Goggins WB. An educational intervention to improve women’s ability to Cope with childbirth. J Clin Nurs. 2009;18:2125–35. 19583645 10.1111/j.1365-2702.2008.02720.x

[49] Effati Daryani F, Mohammadi A, Mirghafourvand M. Childbirth self-efficacy and fear of childbirth and their predictors in adolescent and adult pregnant women referring to health centres of Urmia-Iran: a cross-sectional study. BMJ Open. 2023;13:e077043. 37848306 10.1136/bmjopen-2023-077043 PMC10582945

[50] Uldal T, Østmoen MS, Dahl B, Røseth I. Women’s experiences with hypnobirth – A qualitative study. Sex Reprod Healthc. 2023;37:100890. 37541096 10.1016/j.srhc.2023.100890

[51] Abdelaziz EM, Alsadaan N, Alqahtani M, Elsharkawy NB, Ouda MMA, Ramadan OME, et al. Effectiveness of cognitive behavioral therapy (CBT) on psychological distress among mothers of children with autism spectrum disorder: the role of Problem-Solving appraisal. Behav Sci (Basel). 2024;14:46. 38247698 10.3390/bs14010046 PMC10813282

[52] Dekel S, Papadakis JE, Quagliarini B, Pham CT, Pacheco-Barrios K, Hughes F, et al. Preventing posttraumatic stress disorder following childbirth: a systematic review and meta-analysis. Am J Obstet Gynecol. 2024;230:610–e64114. 38122842 10.1016/j.ajog.2023.12.013 PMC11168224

[53] Lopes MI, Vieira M, Cardoso A. Women’s empowerment for active labor: A qualitative study with nurse-midwives in antenatal education for childbirth. Eur J Midwifery. 2024;8:1–10. 10.18332/ejm/188117 PMC11339881 39175493

[54] Miranda AR, Barral PE, Scotta AV, Cortez MV, Soria EA. An overview of reviews of breastfeeding barriers and facilitators: analyzing global research trends and hotspots. Glob Epidemiol. 2025;9:100192. 40129756 10.1016/j.gloepi.2025.100192 PMC11931314

[55] Islam KF, Awal A, Mazumder H, Munni UR, Majumder K, Afroz K, et al. Social cognitive theory-based health promotion in primary care practice: A scoping review. Heliyon. 2023;9:e14889. 37025832 10.1016/j.heliyon.2023.e14889 PMC10070720

[56] Alruwaili AN, Alruwaili M, Ramadan OME, Elsharkawy NB, Abdelaziz EM, Ali SI, et al. Compassion fatigue in palliative care: exploring its comprehensive impact on geriatric nursing well-being and care quality in end-of-life. Geriatr Nurs (Minneap). 2024;58:274–81. 10.1016/j.gerinurse.2024.05.024 38843756

[57] Mohamed H, Ismail A, Sutan R, Rahman RA, Juval K. A scoping review of digital technologies in antenatal care: recent progress and applications of digital technologies. BMC Pregnancy Childbirth. 2025;25:153. 39948493 10.1186/s12884-025-07209-8 PMC11827299

[58] S J S. Examining socioeconomic factors influencing maternal health in pregnancy. J Hum Behav Soc Environ. 2025;35:469–87.

[59] Xu Q. The impact of new media technology applications on educational equity in rural areas. Educ J. 2024;13:284–93.

