# Supplementary material for: The effectiveness of nurse-led antenatal education on maternal self-efficacy: an evidence-based approach
Source: BMC Nurs. 2025 Jul 10;24:895. doi: 10.1186/s12912-025-03471-5 (PMC12243129; doi:10.1186/s12912-025-03471-5)
Supplement: Supplementary file 1 — Supplementary Material 1 [file 12912_2025_3471_MOESM1_ESM.docx]

Extraction Table Of The Included Studies

| Study | Design | Country | Setting | Sample Size | Maternal Characteristics | Intervention Structure | Educator Background | Content Topics | Instructional Methods | Primary Outcome Measure | Timing of Assessment | Results Summary | Mechanism of Impact |
| --- | --- | --- | --- | --- | --- | --- | --- | --- | --- | --- | --- | --- | --- |
| Brixval et al.[40] | RCT | Denmark | Public maternity hospitals | 1766 | Mixed parity; general population; GA not reported | Small-group sessions (3 x 2.5 hrs) | Midwives | Labor stages, pain coping, birth planning | Group discussion, printed materials | 3 confidence items (non-standardized) | Post-intervention | Higher confidence in labor handling vs auditorium group (p<0.05) | Personal attention, interactive learning increased confidence |
| cáankaya [41] | RCT | Turkey | University hospital | 112 | Primigravidas; aged 18‚Äì35; 20‚Äì28 weeks gestation | 8 sessions (2 hr x 2/wk) | Midwives | Labor stages, pain relief, emotional prep, postpartum care | Slides, relaxation practice, role play | Childbirth Self-Efficacy Inventory (CBSEI) | Pre and post intervention | Significant improvement in CBSEI; reduced fear & anxiety (p<0.05) | Emotional prep, repeated practice, midwife trust |
| AlSomali et al.[42] | RCT | Saudi Arabia | Government hospital clinics | 94 | Primigravidas, age ~26; GA ~28 weeks | Structured program, weekly sessions (not detailed) | Nurses | Stages of labor, breathing, birth plan, postpartum | Visual aids, lectures, Q&A | CBSEI (Arabic version) | Baseline and 36 weeks gestation | CBSEI score improved significantly (mean ‚Üë ~39 points; p<0.001) | Knowledge empowerment, coping skills |
| Frankham et al.[43] | RCT | Australia | Online community-based setting | 125 | Pregnant women, mixed parity, late 2nd to early 3rd trimester | 3-arm trial: She Births¬Æ online course vs booklet vs no intervention | Childbirth educators | Labor stages, emotional prep, breathing, partner support | Video modules, storytelling, interactive discussion forums | Childbirth Self-Efficacy Scale | Pre and post intervention | Online course showed non-significant trend toward higher self-efficacy; no PTSD difference | Knowledge, empowerment, connection via digital peer support |
| Gau et al.[44] | RCT | Taiwan | Teaching hospital maternity unit | 76 | Primigravidas, ~38 per group; third trimester | Birth ball education (prenatal + use during labor) | Nurses and midwives | Labor positions, pain relief, breathing, birth process | Demonstration, hands-on practice, use during labor | CBSEI (Chinese version) | During labor | Significantly higher self-efficacy and lower pain intensity in intervention group | Physical preparedness, improved coping skills |
| Byrne et al.[29] | Quasi-experimental | Australia | Community health centers | 18 | Pregnant women; third trimester; average age ~30 | Mindfulness-Based Childbirth Education, 8 weekly sessions | Certified nurse-midwives | Mindfulness, labor coping, fear reduction, breathing | Guided meditation, group discussion, cognitive skills | CBSEI | Pre and post intervention | Significant increase in self-efficacy; reduced fear of childbirth | Mindfulness enhances coping, trust in self and body |
| osbir et al.[30] | Quasi-experimental | Turkey | Hospital-based classes | 90 | Mixed parity; second trimester enrollment | Multi-week antenatal classes, hospital-based | Nurses and midwives | Labor stages, coping, postpartum care, emotional support | Lectures, demonstrations, group practice | CBSEI, W-DEQ (Fear), Impact of Event Scale (PTSD) | Pre and post intervention | Improved self-efficacy and reduced fear and PTSD symptoms | Increased knowledge and emotional reassurance |
| Sunay [31] | RCT | Turkey | Family Health Center | 153 | Primigravidas, 18-35 years, 28-32 weeks GA | 2√ó45 min nurse-led sessions; one arm had birth plan | Nurses | Labor stages, breathing, birth plans | Discussion, planning exercise, visual aids | CBSEI (Turkish version) | Pre/post intervention | Classes improved CBSEI significantly vs control; no added benefit of birth plan | Knowledge enhancement and emotional reassurance |
| Zaman [24] | Quasi-experimental | Turkey | University hospital | 63 | Pregnant couples; 28-32 weeks GA | Group sessions over 4 weeks | Midwives and nurses | Fear reduction, coping with labor, postpartum care | Lectures, couple interaction | CBSEI, W-DEQ (fear) | Post-intervention | Increased CBSEI scores and reduced fear in class group | Peer support, shared learning with partners |
| Khademioore et al.[32] | RCT | Iran | Primary health center | 70 | Primigravidas, 28-34 weeks GA | 8 weeks tele-midwifery via mobile app | Midwives | Labor stages, coping, partner support | Mobile app, weekly nurse contact | CBSEI, W-DEQ | Pre/post intervention | Improved CBSEI scores and reduced fear; lower cesarean rate | Continuous support, remote reassurance |
| Howarth & Swain[20] | RCT | New Zealand | Home-based (self-guided) | 182 | First-time mothers, 24-36 weeks GA | Pink Kit home program (manual, video, skills) | Self-led, nurse-developed | Labor anatomy, pain coping, emotional prep | Video/manual instruction | CBSEI (NZ version) | Baseline and at 36 weeks | Significant CBSEI improvement in intervention vs control | Hands-on practice improved sense of control |
| Abbasi et al.[33] | RCT | Iran | Teaching hospital | 153 | Primigravidas, 20-28 weeks GA | e-Learning vs booklet vs routine care | Nurses and IT educators | Labor stages, breathing, decision-making | App-based education, booklets | CBSEI | Baseline and during active labor | CBSEI higher in both intervention groups; e-learning most effective | Interactive tools increased confidence |
| Vasegh Rahimparvar et al.[34] | RCT | Iran | Clinic-based | 120 | Primigravidas, ~20-30 weeks GA | CD-ROM based childbirth preparation | Nurses and midwives | Stages of labor, relaxation, delivery positions | Multimedia CD-ROM modules | CBSEI | Pre/post intervention | Significantly higher CBSEI in intervention group | Access to repeated information supports confidence |
| Duncan et al.[35] | RCT | USA | Community-based | 30 | Nulliparas, third trimester | 2.5-day 'Mind in Labor' mindfulness workshop | Nurse-midwives | Mindfulness, breathing, managing labor | Meditation, cognitive reframing | CBSE scale | Pre/post workshop | Greater self-efficacy post-workshop; lower depressive symptoms | Mindfulness fostered emotional control |
| Firouzan et al.[36] | RCT | Iran | Public health center | 80 | Primigravidas with high fear scores | BELIEF counseling (2 in-person + 8 phone) | Midwives | Fear reduction, self-efficacy, vaginal delivery | Cognitive-behavioral approach, phone support | CBSEI, W-DEQ | Pre/post intervention | Improved CBSEI; reduced fear; more preferred vaginal birth | Continuous emotional coaching |
| Nooied et al.[15] | RCT | Thailand | School-linked clinic | 128 | Adolescent pregnant girls | Nurse-led FOC program + mobile app | School nurses | Fear of childbirth, empowerment, labor process | Lectures, app activities, self-tracking | Thai CBSEI, FOC scale | Pre/post | Higher CBSEI and lower fear post-program | Engagement via gamified app, repeated content |
| Youssef et al.[37] | Quasi-experimental | Egypt | Hospital outpatient clinic | 100 | Primigravidas, 28-34 weeks GA | Prenatal childbirth prep classes | Nurses | Labor stages, coping, postpartum care | Visuals, Q&A, group sessions | CBSEI (Arabic) | During first and second labor stages | Greater CBSEI during both labor stages vs control | Practice and preparation supported self-belief |
| Hassan et al.[38] | RCT | Egypt | Maternal hospital clinic | 90 | Primigravidas with fear of childbirth | Nurse-led fear-counseling sessions | Registered nurses | Fear reduction, coping skills, delivery prep | Interactive sessions, birth planning | CBSE, W-DEQ | Pre/post | Significantly reduced fear and higher CBSE in intervention group | Emotional assurance and knowledge-based confidence |
| Rastegari et al.[39] | Quasi-experimental | Iran | Provincial health centers | 60 | Primigravidas, ~28 weeks | Local childbirth preparation sessions | Nurses | Labor knowledge, pain management | Slides, discussions | CBSEI (Persian) | Postpartum (immediate) | Higher CBSEI in class group; not statistically significant | Trend toward improved coping |
| Abd El-Kader[4] | \|  \| \| --- \|  \| Quasi-experimental (non-random) \| \| --- \| | Egypt | Maternal hospital clinic | 148 | women at the end of pregnancy | Childbirth Self-Efficacy Enhancing Classes on Labor Length and Outcomes | Registered nurses | Fear reduction, coping skills, delivery prep | Interactive sessions, birth planning | childbirth self-efficacy inventory, a follow-up checklist for monitoring maternal and neonatal outcomes during labo | Pre/post | Significantly reduced fear and higher CBSE in intervention group | Emotional assurance and knowledge-based confidence |
